# Evaluation of wearing comfort of dust masks

**DOI:** 10.1371/journal.pone.0237848

**Published:** 2020-08-20

**Authors:** Sejin Choi, Ryeol Park, Nahmkeon Hur, Wonjung Kim

**Affiliations:** Department of Mechanical Engineering, Sogang University, Seoul, Republic of Korea; Michigan Technological University, UNITED STATES

## Abstract

Dust masks are widely used to prevent the inhalation of particulate matter into the human respiratory organs in polluted air environments. The filter of a dust mask inherently obstructs the natural respiratory air flows, and this flow resistance is mainly responsible for the discomfort experienced when wearing a dust mask. In atmospheric conditions seriously contaminated with fine dust, it is recommended that common citizens wear a dust mask in their everyday lives, yet many people are reluctant to wear a dust mask owing to the discomfort experienced when wearing it for a long time. Understanding of physical reasons for the discomfort is thus crucial in designing a dust mask, but remains far from clear. This study presents a technique to quantify the wearing comfort of dust masks. By developing a respiration simulator to measure the pressure loss across a dust mask, we assessed the energy costs to overcome flow resistance when breathing through various types of dust masks. The energy cost for a single inhalation varies with the mask type in a range between 0 and 10 mJ. We compared the results with the survey results of 40 people about the wearing comfort of the dust masks, which revealed that the wearing comfort crucially depends on the energy cost required for air inhalation though the dust mask. Using the measured energy cost during inhalation as a parameter to quantify the wearing comfort, we present a comprehensive evaluation of the performance of dust masks in terms of not only the filtering performance but also the wearing comfort. Our study suggests some design principles for dust mask filters, auxiliary electric fans, and check valves.

## Introduction

Particulate matter (PM) inhaled through one’s nose and mouth accumulates in the lungs and airways, resulting in various diseases of the respiratory and cardiovascular systems [1–6]. It was reported that approximately two million people die each year due to diseases associated with air pollution [7]. Health threats caused by PM are still increasing in many countries [8–10], and social concerns about PM have caused international conflicts in East Asia [11].

Dust masks provide a simple yet effective and expedient way of preventing PM inhalation in environmental conditions with a high PM concentration. Dust masks with a wide range of filter classes and various auxiliary attachments such as electric fans or check valves have been developed and marketed in recent years [12,13]. With the rapid growth of the dust mask industry led by East Asian countries, substantial efforts have been devoted to examining the filtering performance of dust masks. Experimental simulators were used to investigate leakage flow through the gap between the mask and facial area for various dust masks [14,15], and human subject experiments were carried out to examine aerosol penetration through dust masks, which raised questions on filtration performance of some dust masks [16,17].

However, filtration performance cannot be the sole factor that represents the performance of dust masks. The purification filter of dust masks is typically made of fabric with a porous structure with micro-sized pores. A filter with smaller pores is advantageous for filtering out either smaller particles or more dust, but it produces a greater flow resistance. In many East Asian countries, recent air pollution has become very serious, and a noxious air condition often lasts for a few weeks. Air Quality Guideline issued by World Health Organization stipulates that the 24-hour mean concentration of particulate matter less than 2.5 microns in diameter (PM 2.5) should be less than 25 μg/m^3^ [18]. However, the annual mean concentrations of PM 2.5 in Beijing, China and New Delhi, India were, for instance, reported to be 52 μg/m^3^ and 140 μg/m^3^, respectively [19,20]. In such atmospheric conditions, it is strongly advised that common citizen wear a dust mask in their everyday lives, but many people refuse to wear a dust mask owing to the discomfort experienced when wearing it for a long time [21]. Therefore, there is a strong demand for dust masks with better wearing comfort in countries with atmospheric conditions seriously contaminated with fine dust. In order to develop a dust mask that alleviates the discomfort caused by wearing the mask, the discomfort detected by wearers must be quantitatively evaluated, but even the cause of the discomfort has been poorly investigated.

In this paper, we present a technique for quantifying the wearing comfort of dust masks. Based on the mechanics of human respiration, we suggest that the additional work required for air inhalation through a dust mask is a direct indicator for measuring the wearing comfort. By developing a respiration simulator, we measured the pressure loss across six dust masks each with a distinct filter class and auxiliary elements such as electric fans or check valves at human breathing conditions, and estimated energetic costs for breathing through the dust masks. The results were compared with the survey scores for wearing comfort obtained from 40 participants in human research. It appears that energy cost, especially during inhalation, is important for comfortability. Using energy cost for inhalation as a parameter for measuring the wearing comfort, we comprehensively evaluated various dust masks in terms of both the filtering performance and the wearing comfort, elucidating how the filter class and additional elements affect the wearing comfort. We end with some suggestions for designing a better dust mask, in particular, with respect to filters, electric fans, and check valves.

## Materials and methods

### Experiments using a respiration simulator

**Fig 1:**
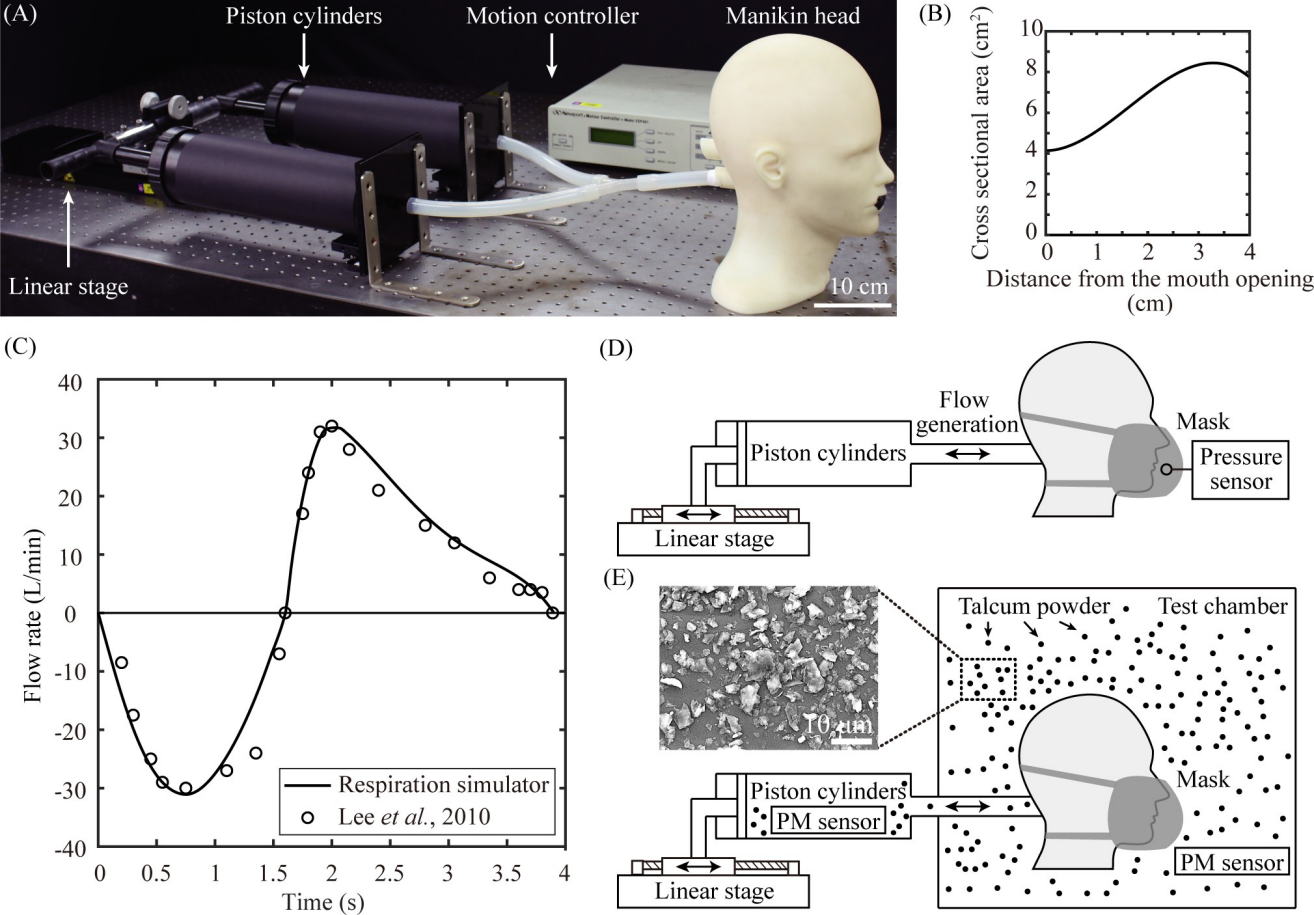
Experimental setup. (A) Respiration simulator consisting of a linear stage, motion controller, piston cylinders, and manikin head. (B) Cross-sectional area of the flow channel in the manikin that varies with the distance from the oral cavity inlet. (C) Flow rate through the air channel in the manikin. The circular symbols represent data measured in human breathing at rest [25]. Schematic illustrations of the experiment setups for measuring (D) the pressure inside the mask cavity and (E) the particle filtration through a mask. The inset shows the SEM image of the talcum powder.

We constructed a respiration simulator that creates an air flow at standard human breathing conditions. As shown in Fig 1A, the system consisted of a linear stage, motion controller, manikin head, and two piston cylinders. Each piston cylinder with an inner diameter of 19.2 cm was connected with a silicone tube with an inner diameter of 55 mm, and the tubes were merged into one with a Y-connector to connect to the manikin head. The two pistons were tied to a single structure to synchronize the motion. The manikin head, made of ABS plastic by machining operations, had the standard physical appearance of Korean men in their 30s with respect to the vertical length and head circumference [22]. An air channel connecting from the rear part to the mouth was created inside the manikin head. The cross-section of the channel was hemi-elliptical, and the cross-sectional area varied depending on the distance from the mouth opening in the same manner as the human airway shown in Fig 1B [23]. Human lips at rest were imitated, so that the mouth opening had an area of 2.2 cm^2^ [24]. A mask under test was worn on the manikin with only the ear strings or with both the ear strings and the head strip, as suggested by the manufacturer.

The motion of the piston cylinders was operated by a linear stage (ESP-301G, NEWPORT), which created an air flow through the channel formed in the manikin head. We controlled the volumetric flow rates over time to be the same as human breathing at rest by controlling the instant speed and acceleration of the linear stage. Fig 1C shows the flow rates generated by our simulator for a single breathing cycle starting with inhalation, which was consistent with the flow rate of human breathing at rest [25]. The flow rate was estimated as the product of the piston speed and the cross-sectional area after we had verified the flow rate through direct measurement of the flow rate with a flowmeter (M-Flowmeter, ALICAT).

Fig 1D illustrates the experimental setup to measure the flow resistance across a dust mask. We measured the pressure difference across a mask put on the manikin head using a pressure gauge (DPG409, OMEGA ENGINEERING). The pressure gauge connected with a silicone tube with an inner diameter of 2 mm was placed 3 cm ahead of the mouth opening. The tube opening was aligned in a direction perpendicular to the air flow to measure the static pressure rather than the dynamic pressure.

Fig 1E shows the experimental setup to measure the dust filtration performance of a mask. In a cubic acrylic chamber with a volume of 75 cm^3^, we dispersed talcum powder (ISNP-3000C, ILSHIN MATERIALS) with an inherent material density of approximately 2.7 g/cm^3^ to simulate an environment polluted with PM. The talcum powder was percolated through a sieve with 10 μm pores, so that the particle size was less than 10 μm (see the inset of Fig 1E). A PM sensor (PMS-7003, PLANTOWER) was placed in the test chamber and the piston cylinder, respectively, to measure the concentration of particles with a size of less than 2.5 μm (PM 2.5). The PM concentration was kept at 75±8 μg/m^3^ in the test chamber during the test. The PM concentration in the cylinder was measured 90 sec after the simulator begun to operate. Although the PM concentration changed little during the measurement period, we took a time average of the PM concentration measured for 10 sec.

### Dust masks

**Fig 2:**
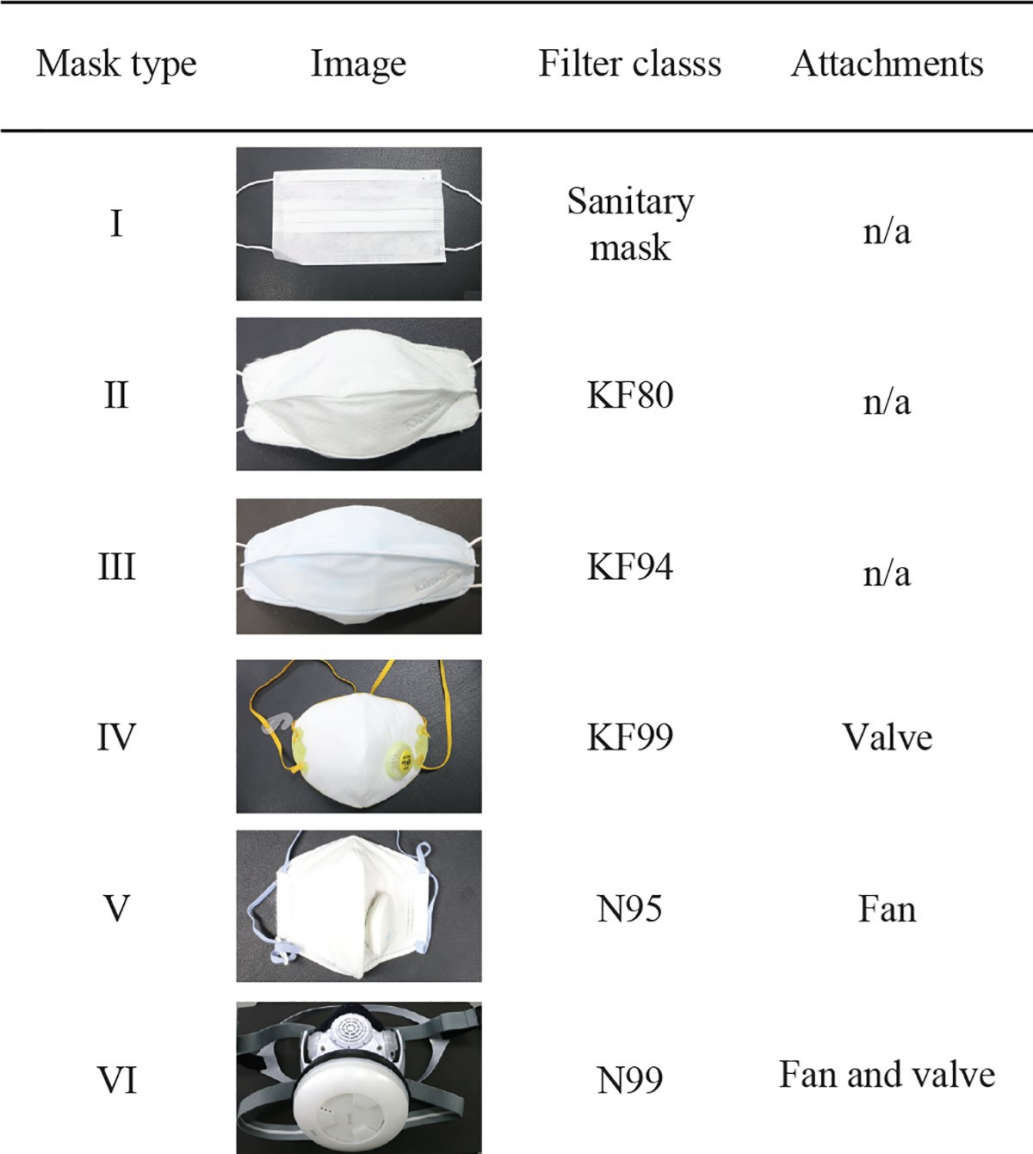
Dust masks tested in the experiment.

We tested six masks available on the market in South Korea listed in Fig 2. The masks varied in terms of filter class and auxiliary elements such as electric fans or check valves. Mask I is typically used for sanitary purposes to prevent spitting out, not for dust filtration, but we used it for comparison with the other dust masks. Masks II-VI are dust masks with a filtration efficiency certified by public organizations. Masks II, III, and IV have the KF80, KF94, and KF99 filter classes, respectively, which are approved by the Korean Food and Drug Administration (KFDA). Masks V and VI have the N95 and N99 filter classes, respectively, approved by the National Institute for Occupational Safety and Health (NIOSH). It was known that the KFDA KF94 and KF99 filter classes are very close to the NIOSH N95 and N99 filter classes, respectively [14,16]. Masks IV-VI have additional elements such as a check valve and electric fan. Mask IV has a check valve on the outer surface of the mask, which opens during the exhalation period. Mask V has an electric fan that generates a consistent air flow from the mask cavity to the surrounding to assist with exhalation. Mask VI has both an electric fan and an exhalation check valve. Contrast to Mask V, the electric fan of Mask VI generates an air flow in the mask cavity from the surrounding and thus assists with inhalation, and the flow rate is controlled depending on the pressure in the mask cavity in a way that the electric fan speed is greatly reduced during exhalation. In addition, Mask VI has a rubber seal along the mouth cavity perimeter to limit air flow through the gap between the mask and the head.

### Survey of mask wearing comfort

We conducted a survey of how people feel wearing comfort for various dust masks. Twenty male and twenty female experiment participants from 20 to 55 years of age were selected to exclude age- and gender-related biases. All the participants had no respiratory disease and were healthy. The participants were asked to wear the six different dust masks for two minutes each at a rest condition and to rank the ease of breathing with each mask. The test order of the masks for each participant was randomized. The comfortability rank of the six masks was converted into a score by giving 1, 2, 3, 4, 5, and 6 points in order from the best wearing comfort to the worst wearing comfort so that a lower score indicates a better wearing comfort. The *t*-test was performed to confirm the significance of the score difference.

Ethics approval for the human subject survey was obtained from the Research Integrity Committee of Sogang University (SGUIRB-A-1912-53). All participants were provided with a written informed consent and able to withdraw from this study at any time. Any personally identifiable information was not collected.

## Results

### Energy cost for breathing with a dust mask

We propose that the discomfort perceived by a mask wearer depends primarily on the additional energy required for breathing through the dust mask which correlates with flow resistance. We proceed by evaluating the additional energy for breathing through a dust mask. To create fluid flow through a channel, force *F* is exerted on the fluid to overcome resistance including fluid inertia and viscous force. When the force creates a fluid motion with a speed of *U*, the time rate of energy input is given by *W* = *FU*. In the case of the pressure driven flow, the work rate is thus expressed as *W* = Δ*PAU*, where Δ*P* is the applied pressure, and *A* is the cross-sectional area of the channel [26]. Accordingly, one can estimate the work rate to create an air flow for breathing without a mask can be estimated as *W*_0_ = (*P*_in_-*P*_out_)*Q*, where *P*_in_ is the intrapleural pressure, *P*_out_ is the atmospheric pressure, and *Q* is the outward flow rate through the airways. With this sign convention, the work rate remains positive throughout natural respiration. For a given human airway system with a specific flow resistance, the pressure difference *P*_in_-*P*_out_ can be expressed in terms of *Q*, so that it can be assumed that *Q* exclusively determines *W*_0_. When one breathes through a mask, the work rate *W*_m_ increases due to the flow resistance produced by the dust mask. In this case, the work rate can be separated into two terms: (*P*_in_-*P*_c_)*Q+*(*P*_c_-*P*_out_)*Q* with *P*_c_ being the pressure in the mask cavity. The first term corresponds to the work rate for overcoming the resistance of the airways, which can be assumed to be the same as *W*_0_ for a specific *Q*. Accordingly, the additional work rate required when one breaths with a mask is given by (*P*_c_-*P*_out_)*Q*. For a breathing time of *T*, the energy cost required to overcome the resistance of a dust mask is therefore calculated to be ∫_*T*_(*P_c_−P_out_*)*Q*d*t*.

**Fig 3:**
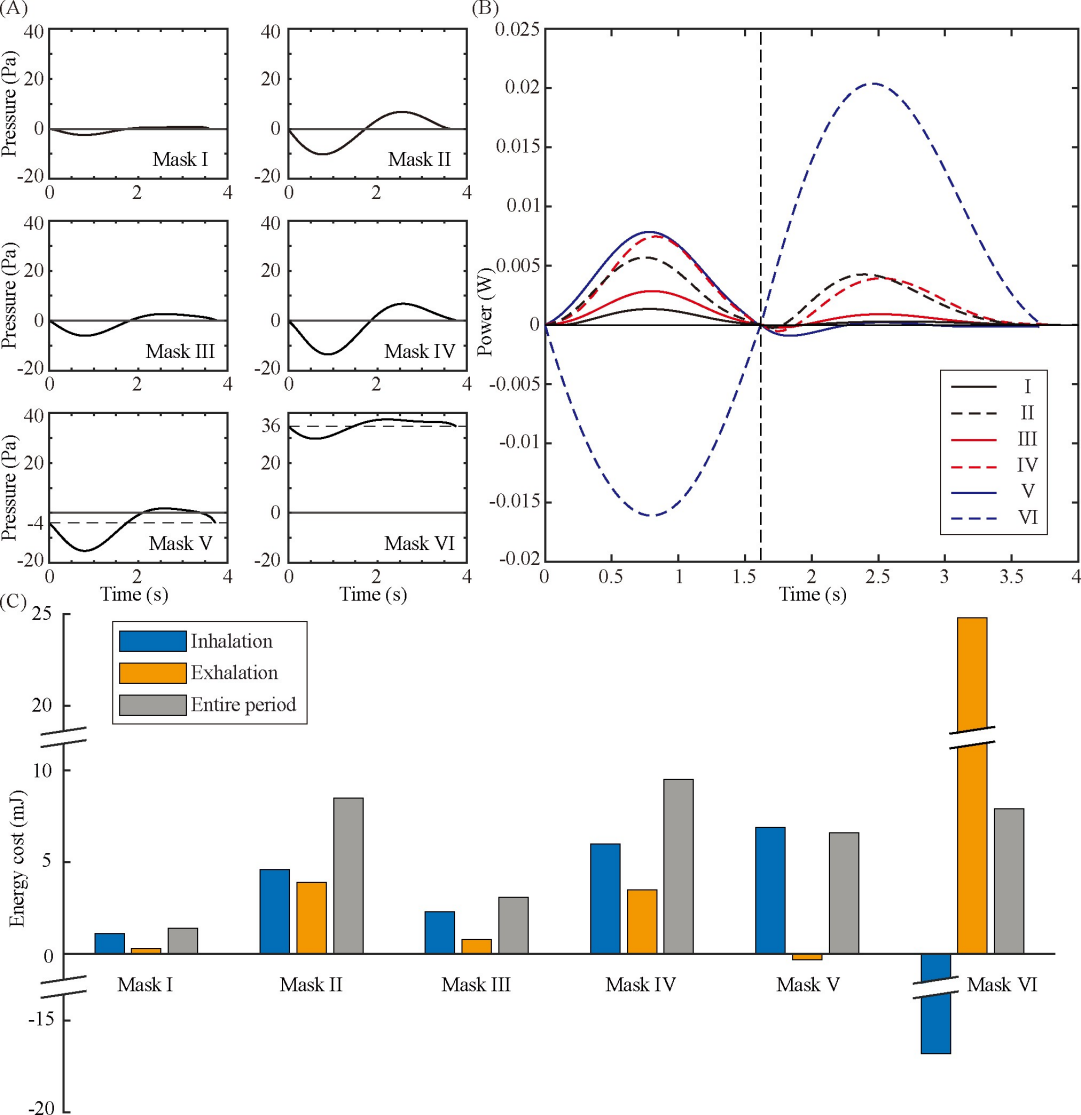
Measurement of pressure in the mask cavity and estimation of energy cost. (A) Pressure measured inside the mask cavity of Masks I–VI. (B) Work rate required for creating an airflow through the dust masks. (C) Energy cost for overcoming the flow resistance of the dust masks during the inhalation, exhalation, and entire respiration periods.

We assessed the work rate using our respiration simulator measuring *P*_*c*_ and *Q*. Fig 3A displays the pressure *P*_*c*_ of the six masks during a single respiration period. For Mask I–IV, the pressure in the mask cavity changes from negative in inhalation to positive in exhalation, so that the pressure curve appears to fluctuate around the axis representing *P*_c_ = 0. For Masks V and VI, the electric fan makes a change in the internal pressure at the beginning of breathing to *P*_c_ = -4 Pa and 36 Pa, respectively, around which the pressure fluctuations occur.

Fig 3B shows the work rate required for creating the air flow through a dust mask, calculated as the product of the pressure and flow rate. Mask I exhibits the lowest work rate, suggesting the lowest flow resistance of the tested masks. Mask I, used not for dust filtration but for preventing spitting out, has a large gap between the manikin head and the mask, enabling a significant amount of air flow to bypass the mask. The small flow resistance of Mask I leads to the relatively small work rate. For Masks II-IV, a mask with a higher filtration class has a higher flow resistance, and the work rate thus increases with the filtration class. Mask IV has the highest filtration class, but the work rate for exhalation is similar to the work rate of Mask II, thanks to the check valve at work during exhalation. The electric fans of Masks V and VI yield positive work done on the simulator, and the work rate required for breathing is reduced and temporarily records negative values. For Mask V, the electric fan creates an air flow outward, which thus raises the work rate during inhalation and reduces the work rate during exhalation. The power curve of Mask VI with both a check valve and an electric fan seems sinusoidal with a large amplitude apparently distinct from the others. The powerful electric fan attached to Mask VI is responsible for the large amplitude of the power curve. The electric fan creating an air flow inward during inhalation renders the work rate negative for the inhalation period. Due to the check valve that opens at a pressure above the cracking pressure, the pressure inside the mask cavity remains higher than atmospheric pressure, leading to a high resistance over exhalation.

Taking the integral of the work rate over time yields the energy cost for breathing with a dust mask. Fig 3C shows the energy costs for the six dust masks for a single inhalation and a single exhalation periods and for a single breathing period.

### Quantification of wearing comfort of the dust masks

**Fig 4:**
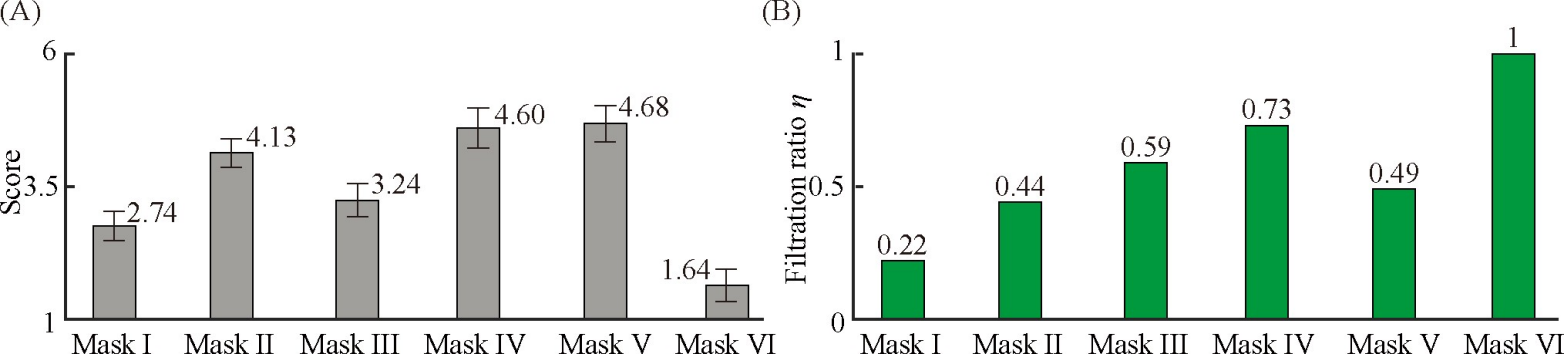
Wearing comfort scores and filtration ratio. (A) Wearing comfort scores of the dust masks from the survey of 40 participants. The comfortability rank of the six masks in order from best to worst was converted into scores by giving 1, 2, 3, 4, 5, and 6 points. The error bars indicate the 95% confidence interval. (B) Filtration ratio of the dust masks defined as η = (C_o_—C)/C_o_. Filtration ratios of 0 and 1 correspond to no filtering action and perfect filtration, respectively.

We examined how the energy cost affects the wearing comfort. Fig 4A shows the scores of the six masks which were obtained from the survey of the 40 participants in the human research. The score ranges from 1 to 6 in the direction of the best to the worst wearing comfort. The participants appreciated the comfortability in the order of Mask VI, I, III, II, IV, and V. There is significant difference of the score from Mask VI to Mask IV, as confirmed by the results of *t*-tests between two masks: for Mask VI (M = 2.74, SD = 1.17) and Mask I (M = 1.64, SD = 1.38), *t*(DOF = 77) = 4.36, *p* < 0.0005; for Mask I and Mask III (M = 3.24, SD = 1.42), *t*(77) = -1.97, *p* < 0.054; for Mask III and Mask II (M = 4.13, SD = 1.05), *t*(76) = 3.57, *p* < 0.0007; for Mask II and Mask IV (M = 4.6, SD = 2.08), *t*(70) = -1.70, *p* < 0.094. Note that for Mask IV and Mask V (M = 4.68, SD = 1.65), *t*(77) = -0.25, *p* < 0.81, showing no significant difference between the survey scores. Note that Mask VI has the lowest score despite the highest filter class, whereas Mask IV with a check valve and Mask V with an electric fan have the highest scores. This suggests that the auxiliary elements do not necessarily assure a sufficient level of comfortability.

Comparing the survey score with the energy costs, we found that the order of the low survey scores was in a complete agreement with the order of the low energy costs during inhalation. This result reveals that wearing comfort is crucially associated with the energy cost for inhalation and therefore suggests the energy cost for inhalation as a parameter to quantify the wearing comfort. It is noteworthy that the energy cost of Mask VI for the exhalation period is remarkably high, yet its wearing comfort score is the lowest. This signifies that the participants are not sensitive to the energy cost for exhalation, despite the lack of physiological explanations which is beyond the scope of the present study.

### Dust filtering performance

We proceed by analyzing the dependence of wearing comfort on the filtering performance of the dust masks. We quantify the filtering performance using a PM filtration ratio defined as *η* = (*C*_*o*_—*C*)/*C*_*o*_, where *C* and *C*_*o*_ are the talcum powder concentrations measured in the cylinder of the respiratory simulator with a mask and without a mask, respectively. Since some talcum particles can adhere to the inner surface of the channel in the manikin and the cylinder during transport, *C*_*o*_ was measured to be 41±5 μg/m^3^, lower than the surrounding concentration 75±8 μg/m^3^. The filtration ratios of 0 and 1 correspond to no filtering action and perfect filtration, respectively.

Fig 4B shows the assessment of *η* for the six tested masks. Mask I has the lowest protection level against PM 2.5. The experimental results for the other dust masks show that the filtering ratio generally increases with the filter class, suggesting that the filter class is the key factor to determine *η*. However, the dependence of *η* on the energy cost for inhalation is found to be elusive. For instance, Mask VI uniquely has the lowest energy cost during inhalation despite having the highest *η* because the energy cost caused by the filter can be compensated for by the electric fan.

## Discussion

Quantitative measurements of both wearing comfort and filtering ratio enable a comprehensive comparison of the tested dust masks. We here summarize the comparative characteristics of the dust masks. Mask I achieves a good breathability due to the large amount of air flow leaking through the gap, which however results in a low filtration ratio. For Masks II-IV, a dust mask with higher filter class has a relatively better filtration ratio, but less wearing comfort. Mask V does not have a good wearing comfort nor filtration ratio despite the high filter class and an auxiliary fan. Mask VI offers markedly a superior filtration ratio and wearing comfort thanks to the proper operation of the inhalation-assist fan and check valve.

**Fig 5:**
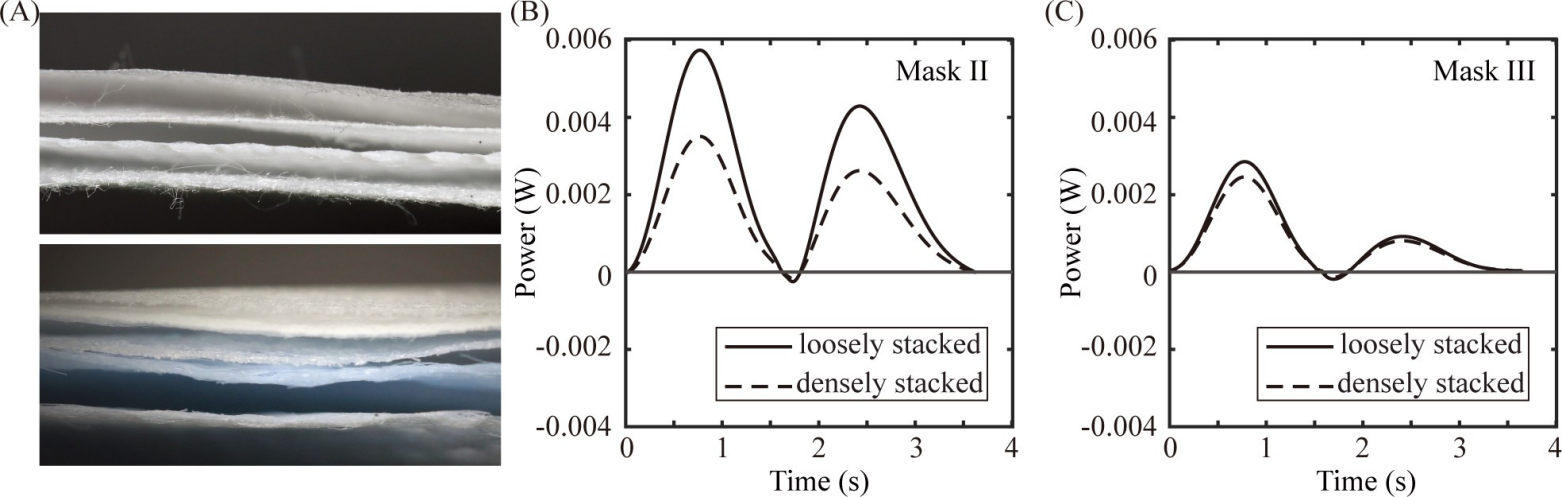
Effects of the filter stack density on the energy cost. (A) Cross-sectional images of the filter layers of Mask II (top) and Mask III (bottom). Comparison of the additional power between loosely stacked and densely stacked cases for (B) Mask II and (C) Mask III.

As seen by Masks II-IV, there is a trade-off between filtration ratio and wearing comfort. The relative importance between the two factors may differ depending on the air quality of the place of use or the preference of the user. Nevertheless, the present results suggest some general design principles for dust mask filters, auxiliary fans, and check valves. First, a dust mask can have a better filtration ratio without losing wearing comfort by having a proper filter structure, exemplified in the observation that a filter with compact layers is advantageous for wearing comfort. Compared with Mask III (KF94), Mask II (KF80) has a lower filter class and as a result, has a lower filter ratio, but it has a greater energy cost for inhalation. We ascribe this to the relatively loosely stacked filter layers of Mask II. We observed that the volume between the filter layers of Mask II expands and shrinks depending on the flow direction in a single respiratory cycle. Such deformation of the filters involves internal air flow in the volume between the layers, which supposedly results in the additional flow resistance. Fig 5 shows that the energy cost for inhalation indeed decreased when the layers were compactly stacked using a staple. Mask II showed a 39% decrease in the energy cost compared to a loosely stacked case, whereas Mask III showed only a 13% decrease.

**Fig 6:**
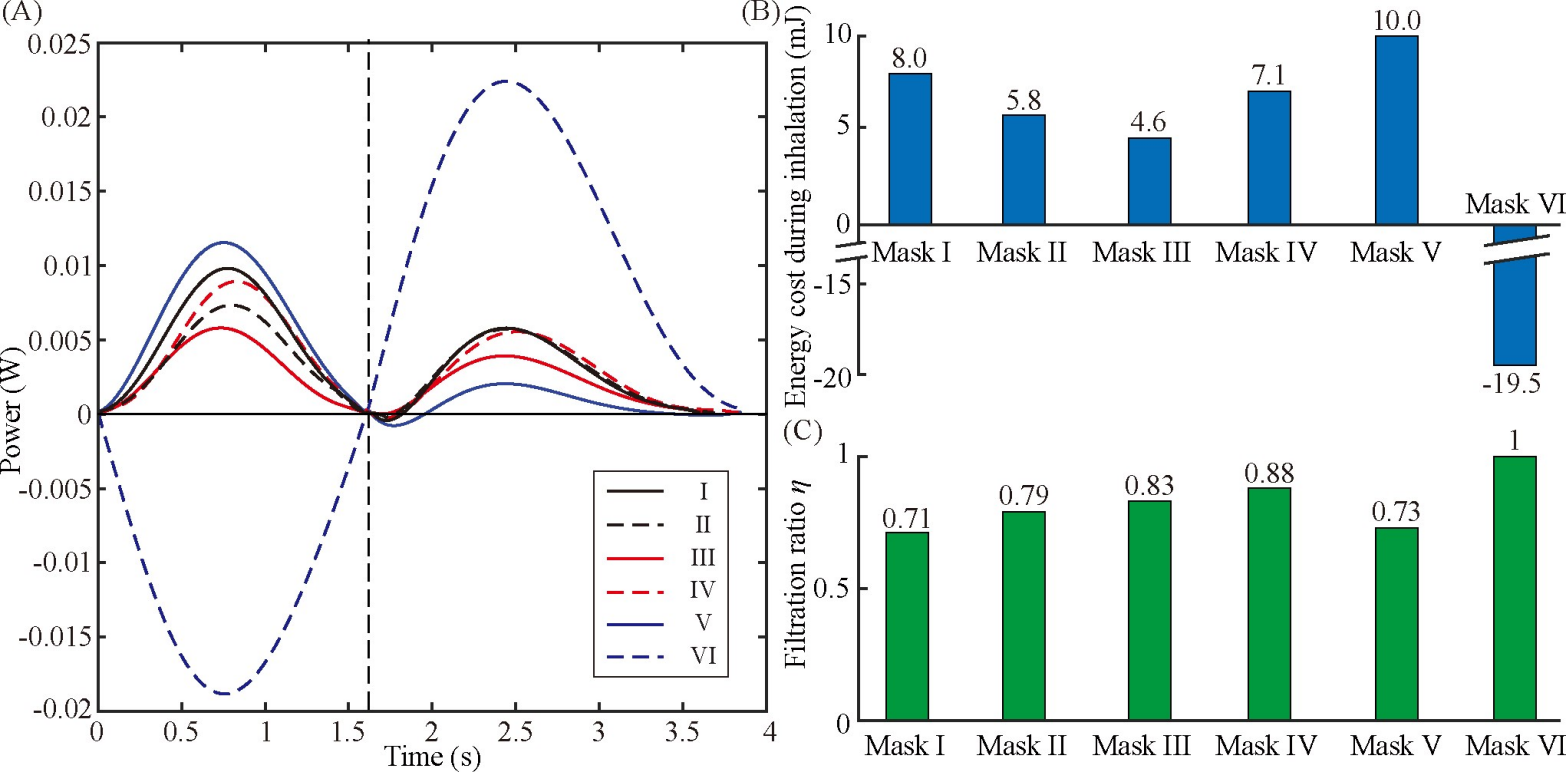
Measurement of the energy cost and filtration ratio in the tightly wearing condition. (A) Work rate required for creating an airflow through the dust mask. (B) Energy cost for overcoming the flow resistance of the dust masks during the inhalation. (C) Filtration ratio of the dust masks (η).

Our experimental results show that air flow through the gap between the mask and the manikin head crucially affects the filtration ratio. Since the KF99 filter class is comparable with the N99, the filtration ratios of Mask IV and VI are expected to be similar to each other, but a significant difference was observed. We speculate that the method of wearing a tightly fitted mask with a rubber seal around the mask cavity minimizes the flow through the gaps other than the filter in Mask VI, thereby yielding a virtually perfect filtering ratio. To quantify the effects of a leakage flow through the gap between the mask and the manikin head on filtration ratio and wearing comfort, we conducted additional experiments to measure the energy cost and the filtration ratio of the masks when an airtight seal limits a leakage flow. Fig 6A presents the work rates required for creating an air flow through a tightly fitted dust mask. As a consequence of the restriction of the leakage flow, the energy costs increase compared to those under the normal wearing condition shown in Fig 3B. Fig 6B shows the energy costs during inhalation for the tightly fitted mask, which reveals the significance of the leakage flow for each mask. The airtight seal also influences the filtration ratio, as demonstrated in Fig 6C. The filtration efficiency is significantly improved compared to the normal wearing condition for all the masks except for Mask VI that has a rubber seal. In particular, Mask I used for sanitary purpose exhibits a comparable filtration ratio to other dust masks in the tightly fitted condition, but also exhibits a significant increase (~600%) in the energy cost during inhalation. This demonstrates the trade-off between the filtration ratio and the wearing comfort, thus suggesting that filtration ratio cannot be the only factor to consider in developing a dust mask.

One notes that the check valve of Mask VI keeps the pressure of the mask cavity higher than the surrounding. As a result, the check valve is also advantageous for preventing a leaking flow inward (see Fig 3A). There are, however, caveats to the use of check valves. Although the cut-off pressure of a check valve higher than atmospheric pressure can be exploited, an excessively high cutoff pressure can induce an unnecessary energy cost during exhalation. For instance, Mask VI can save energy cost for exhalation by reducing the cut-off pressure in such a way that the minimum pressure during inhalation is just above atmospheric pressure.

**Fig 7:**
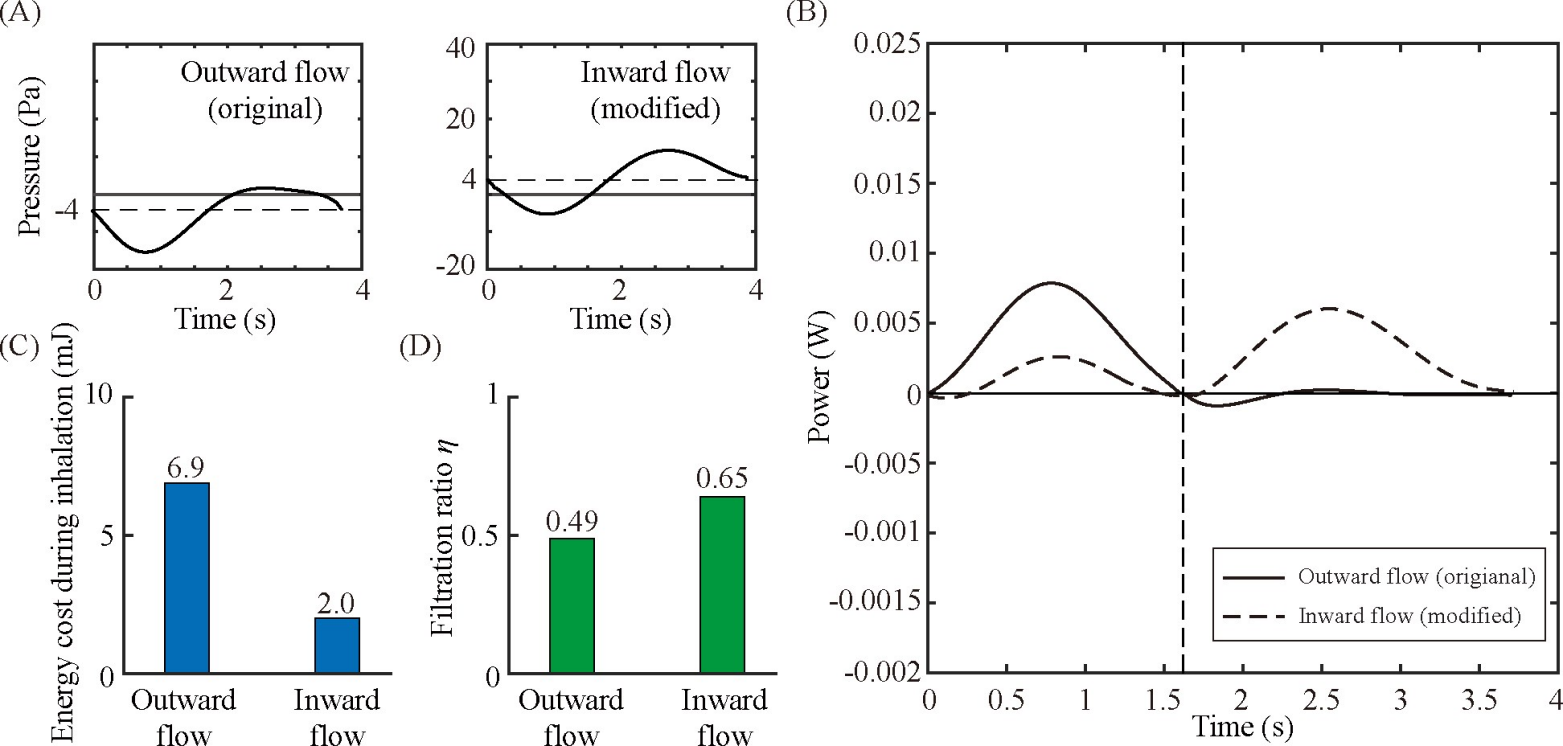
Effects of the flow direction of an auxiliary fan of Mask V. (A) Pressure measured inside the mask cavity of Masks V with a fan creating outward air flow (original Mask V) and inward air flow (modified Mask V). (B) Work rate required for creating an air flow through the dust mask. (C) Energy cost for overcoming the flow resistance of the dust masks during the inhalation. (D) Filtration ratio of the dust masks (η).

Finally, the proper operation of the electric fan attached to a dust mask is described. Both Masks V and VI have an electric fan, but the flow direction and fan power differ for each other. While the fan of Mask V creates a gentle outward flow, the fan of Mask VI creates a powerful inward flow. We propose that an auxiliary fan creating an inward flow is better not only for reducing the leakage but also for improving the wearing comfort. Mask V has the highest energy cost during inhalation despite its relatively low filtration ratio (N95). Moreover, compared with Mask III (KF94), Mask V exhibits a considerably lower filtration ratio but higher energy cost for inhalation. This is most likely due to the electric fan that creates an outward flow. The fan reduces the pressure in the mask cavity during inhalation, resulting in an increase of the energy cost. To demonstrate the effect of the flow direction, we conducted additional experiments by simply modifying the fan direction in a way to reverse the flow direction of Mask V (see Fig 7). The fan on Mask V originally generates the outward air flow through the mask to assist exhalation, but the modified fan now creates inward airflow with the same electric power, leading to increase in the internal pressure. Therefore, the energy cost during inhalation is reduced, and one expects a better wearing comfort (Fig 7C). A raise in the internal pressure is also advantageous for limiting a leakage flow, and as a result improves the filtration ratio, as shown in Fig 7D [12]. In addition, we discuss the desirable power level of the electric fan. Zero energy cost during inhalation corresponds to breathing without a dust mask, and negative work can indicate forced inhalation. Accordingly, we conjecture that excessively negative work can produce an unpleasant feeling, and a fan power equal to the energy cost for breathing through the dust mask is thus most desirable for wearing comfort and for saving electric energy.

## Conclusions

By developing a respiration simulator, we have measured the pressure loss while breathing through six distinct dust masks and estimated the energy cost for breathing through the dust masks. In the comparison of the results with a survey of 40 participants on wearing comfort, we have found that the wearing comfort of dust masks is mainly determined by the energy cost during inhalation. Utilizing the energy cost during inhalation as a quantifiable parameter has led us to a comprehensive evaluation of the dust masks in terms of both filtering performance and wearing comfort. The results have shown that the filtering ratio can increase from 0.22 (Mask I) to 0.73 (Mask IV) with the filter class at the expense of wearing comfort, and additional elements can improve the wearing comfort, but only under certain operation conditions. Therefore, the proper combination of filter grade and additional elements is crucial to reduce the energy cost for inhalation and improve wearing comfort. We have suggested an improved design of dust masks, in terms of mask filters, electric fans, and check valves. Our study provides not only a better physical understanding of breathing through a dust mask but also design guidelines for dust masks to improve both the wearing comfort and filtering performance. Although the present work has focused on the breathing discomfort of a dust mask, the comfort level of the wearer can be also affected by the relative humidity and ear pain by the ear straps. Further studies on the effects of humidity in the mask cavity and stresses on the ears will be useful for better quantifying the wear comfort of dust masks.

## References

[1] Atkinson RW, Fuller GW, Anderson HR, Harrison RM, Armstrong B. Urban ambient particle metrics and health: a time-series analysis. Epidemiology. 2010;21(4): 501–511. 10.1097/EDE.0b013e3181debc88 20502338

[2] Cadelis G, Tourres R, Molinie J. Short-term effects of the particulate pollutants contained in Saharan dust on the visits of children to the emergency department due to asthmatic conditions in Guadeloupe (French Archipelago of the Caribbean). PloS ONE. 2014;9(3): e91136 10.1371/journal.pone.0091136 24603899 PMC3946322

[3] Correia AW, Pope CA III, Dockery DW, Wang Y, Ezzati M, Dominici F. The effect of air pollution control on life expectancy in the United States: an analysis of 545 US counties for the period 2000 to 2007. Epidemiology. 2013;24(1): 23–31. 10.1097/EDE.0b013e3182770237 23211349 PMC3521092

[4] Meister K, Johansson C, Forsberg B. Estimated short-term effects of coarse particles on daily mortality in Stockholm, Sweden. Environmental Health Perspectives. 2012;120(3): 431–436. 10.1289/ehp.1103995 22182596 PMC3295353

[5] Fang Y, Naik V, Horowitz LW, Mauzerall DL. Air pollution and associated human mortality: the role of air pollutant emissions, climate change and methane concentration increases from the preindustrial period to present. Atmospheric Chemistry and Physics. 2013;13(3): 1377–1394.

[6] Löndahl J, Massling A, Pagels J, Swietlicki E, Vaclavik E, Loft S. Size-resolved respiratory-tract deposition of fine and ultrafine hydrophobic and hygroscopic aerosol particles during rest and exercise. Inhalation Toxicology. 2007;19(2): 109–116. 10.1080/08958370601051677 17169858

[7] Shah AS, Langrish JP, Nair H, McAllister DA, Hunter AL, Donaldson K, et al Global association of air pollution and heart failure: a systematic review and meta-analysis. The Lancet. 2013;382(9897): 1039–1048. 10.1016/S0140-6736(13)60898-3 PMC3809511 23849322

[8] Hunt A, Abraham JL, Judson B, Berry CL. Toxicologic and epidemiologic clues from the characterization of the 1952 London smog fine particulate matter in archival autopsy lung tissues. Environmental Health Perspectives, 2003;111(9): 1209–1214. 12842775 10.1289/ehp.6114 PMC1241576

[9] Cheng Z, Jiang J, Fajardo O, Wang S, Hao J. Characteristics and health impacts of particulate matter pollution in China (2001–2011). Atmospheric Environment. 2016;65: 186–194.

[10] Kim KH, Kabir E, Kabir S. A review on the human health impact of airborne particulate matter. Environment International. 2015;74: 136–143. 10.1016/j.envint.2014.10.005 25454230

[11] Kim SE, Bell ML, Hashizume M, Honda Y, Kan H, Kim H. Associations between mortality and prolonged exposure to elevated particulate matter concentrations in East Asia. Environment International. 2018;110: 88–94. 10.1016/j.envint.2017.10.010 29097051

[12] Watanabe M. About a Mask Inner-Pressure Fitting Tester and the Measurement of its Protection Factor. Journal of Science of Labour. 2014;90(2): 71–75.

[13] Cherrie JW, Apsley A, Cowie H, Steinle S, Mueller W, Lin C, et al Effectiveness of face masks used to protect Beijing residents against particulate air pollution. Occupational Environmental Medicine. 2018;75(6): 446–452. 10.1136/oemed-2017-104765 29632130 PMC5969371

[14] Mueller W, Horwell CJ, Apsley A, Steinle S, McPherson S, Cherrie JW, et al The effectiveness of respiratory protection worn by communities to protect from volcanic ash inhalation. Part I: Filtration efficiency tests. International Journal of Hygiene and Environmental Health. 2018;221(6): 967–976. 10.1016/j.ijheh.2018.03.012 29779694

[15] Rengasamy S, Zhuang Z, Niezgoda G, Walbert G, Lawrence R, Boutin B, et al A comparison of total inward leakage measured using sodium chloride (NaCl) and corn oil aerosol methods for air-purifying respirators. Journal of Occupational and Environmental Hygiene. 2018;15(8): 616–627. 10.1080/15459624.2018.1479064 29781773 PMC6198249

[16] Jung H, Kim J, Lee S, Lee J, Kim J, Tsai P, et al Comparison of filtration efficiency and pressure drop in anti-yellow sand masks, quarantine masks, medical masks, general masks, and handkerchiefs. Aerosol Air Quality Research. 2014;14(14): 991–1002.

[17] Steinle S, Sleeuwenhoek A, Mueller W, Horwell CJ, Apsley A, Davis A, et al The effectiveness of respiratory protection worn by communities to protect from volcanic ash inhalation. Part II: Total inward leakage tests. International Journal of Hygiene and Environmental Health. 2018;221(6): 977–984. 10.1016/j.ijheh.2018.03.011 29861400

[18] World Health Organization. WHO Air quality guidelines for particulate matter, ozone, nitrogen dioxide and sulfur dioxide-Global update 2005-Summary of risk assessment, 2006; Available from: https://apps.who.int/iris/handle/10665/69477/

[19] Zhang L, An J, Liu M, Li Z, Liu Y, Tao L, et al Spatiotemporal variations and influencing factors of PM2.5 concentrations in Beijing, China. Environmental Pollution. 2020;262(114276). 10.1016/j.envpol.2020.114276 32179215

[20] Tripathi SN, Lalchandani V, Kumar V, Tobler A, Thambam N, Mishra S, et al. Chemical characterization of fine particulate matter, and source apportionment of organic aerosol at three sites in New Delhi, India. American Geophysical Union Fall Meeting. 2019;

[21] Li Y, Tokura H, Guo YP, Wong ASW, Wong T, Chung J, et al Effects of wearing N95 and surgical facemasks on heart rate, thermal stress and subjective sensations. International Archives of Occupational and Environmental Health. 2005;78(6): 501–509. 10.1007/s00420-004-0584-4 15918037 PMC7087880

[22] Size Korea, Korean Agency for Technology and Standards. Head size measurement data; 2018 [cited 2019 June 26]. Available from: https://sizekorea.kr/measurement-data/head

[23] Cheng KH, Cheng YS, Yeh HC, Swift DL. Measurements of airway dimensions and calculation of mass transfer characteristics of the human oral passage. Journal of Biomechanical Engineering. 1997;119(4): 476–482. 10.1115/1.2798296 9407288

[24] Gupta JK, Lin CH, Chen Q. Characterizing exhaled airflow from breathing and talking. Indoor Air. 2010;20(1): 31–39. 10.1111/j.1600-0668.2009.00623.x 20028433

[25] Lee JH, Na Y, Kim SK, Chung SK. Unsteady flow characteristics through a human nasal airway. Respiratory Physiology & Neurobiology. 2010;172(3): 136–146. 20471501 10.1016/j.resp.2010.05.010

[26] Whites F. Fluid Mechanics. 8th ed McGraw Hill; 2015.

